# Supplementary material for: Sulforaphane reduces obesity by reversing leptin resistance
Source: eLife. 2022 Mar 24;11:e67368. doi: 10.7554/eLife.67368 (PMC8947770; doi:10.7554/eLife.67368)
Supplement: Transparent reporting form [file elife-67368-transrepform1.pdf]

Westbrook Centre
Milton Road
Cambridge CB4 1YG, UK

P 01223 855340
W elifesciences.org
T @elife

eLife's transparent reporting form

We encourage authors to provide detailed information *within their submission* to facilitate the interpretation and replication of experiments. Authors can upload supporting documentation to indicate the use of appropriate reporting guidelines for health-related research (see EQUATOR Network), life science research (see the BioSharing Information Resource), or the ARRIVE guidelines for reporting work involving animal research. Where applicable, authors should refer to any relevant reporting standards documents in this form.

If you have any questions, please consult our Journal Policies and/or contact us: editorial@elifesciences.org.

Sample-size estimation

- You should state whether an appropriate sample size was computed when the study was being designed
- You should state the statistical method of sample size computation and any required assumptions
- If no explicit power analysis was used, you should describe how you decided what sample (replicate) size (number) to use

Please outline where this information can be found within the submission (e.g., sections or figure legends), or explain why this information doesn't apply to your submission:

Experimental sample sizes used are noted in the figure legends. Cohort sizes were determined based on the experience of the co-authors (IC, MGL, and NMR) as well as the published literature of similar experimental protocols. Previous results and analysis of feeding experiments indicate sample sizes that would suffice to observe the statistical significance ($p<0.05$) given a physiologically significant observation. Number of animals used in the metabolic cage measurements were determined based on the available animal housing chambers of the unit in our facilities. RNA sequencing was conducted using 5 samples (5 mice) per cohort based on similar analysis by numerous other groups in the literature.

Replicates

- You should report how often each experiment was performed
- You should include a definition of biological versus technical replication
- The data obtained should be provided and sufficient information should be provided to indicate the number of independent biological and/or technical replicates
- If you encountered any outliers, you should describe how these were handled
- Criteria for exclusion/inclusion of data should be clearly stated
- High-throughput sequence data should be uploaded before submission, with a private link for reviewers provided (these are available from both GEO and ArrayExpress)

Please outline where this information can be found within the submission (e.g., sections or figure legends), or explain why this information doesn't apply to your submission:

eLife Sciences Publications, Ltd is a limited liability non-profit non-stock corporation incorporated in the State of Delaware, USA, with company number 5030732, and is registered in the UK with company number FC030576 and branch number BR015634 at the address Westbrook Centre, Milton Road, Cambridge CB4 1YG.

Majority of the animal experiments in the study were conducted at least in two separate cohorts to confirm the reproducibility of the findings with similar outcomes at different institutions including Vanderbilt University, University of Michigan and Qatar University by different investigators to increase the confidence of the findings and to exclude potential variations that might derive from individual animal facilities. There is no data presented that is known to be irreproducible.
In the metabolic cage studies described in Figure 2, the data collected during the time frame (approximately one hour) the metabolic cages were opened for mouse injections were excluded from analysis. For the studies that involve stereotaxic surgeries into the lateral ventricle, the animals that tested negative during the water intake test (Angiotensin II test) were excluded from the study.

One mouse from the vehicle group in Figure 2G, and 2 mice from the vehicle group and one mouse from the SFN group in Figure 2H were excluded because of the technical errors associated with sensors of the metabolic cages.

One vehicle treated mouse and one SFN treated mouse were excluded from the data presented in Supplementary Figure 5A and 5B. Vehicle treated mouse was excluded due to lethargic symptoms, recommended by the Veterinary, and the SFN-treated mouse was excluded due to failed icv injection. Inclusion of the data from these two mice do not change the statistics or the interpretation of the findings.

There was no animal death or sickness detected that was associated with any of the treatments conducted throughout any of the experiments reported in this study. Animals that died during the experiments/acclimation periods were excluded from the cohorts.

Biological replicates: biologically distinct samples demonstrating the distribution of population. **Technical replicates:** independent measures that indicate the noise associated with the nature of the experiments.

RNA sequencing (raw and processed) data have been uploaded on GEO and datadryad.org and links are attached.

eLife Sciences Publications, Ltd is a limited liability non-profit non-stock corporation incorporated in the State of Delaware, USA, with company number 5030732, and is registered in the UK with company number FC030576 and branch number BR015634 at the address Westbrook Centre, Milton Road, Cambridge CB4 1YG.

Statistical reporting

- Statistical analysis methods should be described and justified
- Raw data should be presented in figures whenever informative to do so (typically when N per group is less than 10)
- For each experiment, you should identify the statistical tests used, exact values of N, definitions of center, methods of multiple test correction, and dispersion and precision measures (e.g., mean, median, SD, SEM, confidence intervals; and, for the major substantive results, a measure of effect size (e.g., Pearson's r, Cohen's d)
- Report exact p-values wherever possible alongside the summary statistics and 95% confidence intervals. These should be reported for all key questions and not only when the p-value is less than 0.05.

Please outline where this information can be found within the submission (e.g., sections or figure legends), or explain why this information doesn't apply to your submission:

> Statistical analysis including mean, variance, and distribution of data, statistical tests used for each comparison, n-values as well as p-values have been indicated in related results section and figure legends.

(For large datasets, or papers with a very large number of statistical tests, you may upload a single table file with tests, Ns, etc., with reference to sections in the manuscript.)

Group allocation

- Indicate how samples were allocated into experimental groups (in the case of clinical studies, please specify allocation to treatment method); if randomization was used, please also state if restricted randomization was applied
- Indicate if masking was used during group allocation, data collection and/or data analysis

Please outline where this information can be found within the submission (e.g., sections or figure legends), or explain why this information doesn't apply to your submission:

> Genotype of mice and/or type of injections (drug vs vehicle) they received determined experimental groups. Beyond that, allocation of samples/animals were random. In most experiments, the investigators were not blinded to the experiment.

Additional data files ("source data")

- We encourage you to upload relevant additional data files, such as numerical data that are represented as a graph in a figure, or as a summary table
- Where provided, these should be in the most useful format, and they can be uploaded as "Source data" files linked to a main figure or table
- Include model definition files including the full list of parameters used
- Include code used for data analysis (e.g., R, MatLab)
- Avoid stating that data files are "available upon request"

Please indicate the figures or tables for which source data files have been provided:

> Excel files have been attached.
